# The Comparison of Self-differentiation and Self-concept in Divorced and Non-divorced Women Who Experience Domestic Violence

**DOI:** 10.5812/ijhrba.10029

**Published:** 2013-09-20

**Authors:** Fatemeh Sheikh, Anahita Khodabakhshi Koolaee, Masoumeh Rahmati Zadeh

**Affiliations:** 1 Department of Psychology, University of Science and Culture, Tehran, IR Iran; 2 Department of Behavioral Sciences, University of Social Welfare and Rehabilitation Sciences, Tehran, IR Iran

**Keywords:** Domestic Violence, Self Concept, Divorce, Female

## Abstract

**Background:**

A number of researches indicate that domestic violence (DV) causes abuse and vulnerability of women and children. Two components that can decrease violence and divorce are self-concept and self-differentiation.

**Objectives:**

In this research, we compare self-differentiation and self-concept in divorced and non-divorced women that experience domestic violence.

**Materials and Methods:**

To achieve the goal of the research, 80 divorced women with domestic violence were chosen through available sampling and equalized with 80 non divorced women with domestic violence in aspect of age and education. They respond to instrument of self-differentiation (Skowron) and self-concept (Rodgers). Data was analyzed between the two groups using independent t-test. The significant level was (P < 0.01).

**Results:**

The findings indicated divorced women have more self-differentiation and self-concept than non-divorced women. In addition, there is a significant difference with respect to self-differentiation and self-concept in divorced and non-divorced women with domestic violence.

**Conclusions:**

These results emphasize that self-differentiation and self-concept can be considered in premarital education (therapeutic interventions) to protective conditions against the occurrence of DV.

## 1. Background

Domestic violence (DV), also known as intimate partner violence (IPV) (1), is defined as the behavior within an intimate relationship that causes physical, sexual, or psychological harm, including the acts of physical aggression, sexual coercion, psychological abuse and controlling behaviors (2-4). Violence against women takes various forms according to different societies and cultures, but its existence is a social fact that is found in all social classes, cultures, religions and geopolitical situations (5, 6). Unfortunately, this means that DV against women is a worldwide phenomenon and therefore, a global public health problem due to the effects it has on women’s health. The invisibility of the phenomenon is due to the fact that women have difficulty in voicing their problems (4, 5, 7). It is known throughout the world that domestic violence is a generalized phenomenon that has negative effects on the life and health of women. Women who have suffered from DV are at higher risk of depression, chronic pain, psychosomatic disturbances, physical injuries, gastrointestinal disorders, posttraumatic symptoms, poor pregnancy outcomes, sense of helplessness and worthlessness , and others (5, 8, 9) which may, in turn, lead to self-destructive behaviors such as smoking, heavy drinking, or suicide. As such, in 1996, the World Health Assembly Resolution (WHA) stated that preventing violence against women is a public health priority (5). Worldwide population surveys among women indicated that between 10% and 50% were, at some stage, abused by an intimate partner (1). Iran shares the widespread social problem of high incidence of DV with developed countries (10). Thirty percent of married women reported at least one act of serious physical violence during their marriage according to a National Survey on DV against Women (NSDV) conducted in 28 provinces of Iran (10). However, it remains invisible and under-reported (7, 10). In all societies, DV is difficult to study because it occurs primarily within the private sphere of the family. Many women are reluctant to report their abuse because of the stigma, self-blame surrounding the experience, fear of possible repercussions from perceived disloyalty to their spouse and family, fear of losing their children, feelings of shame, denial, or fear of being negatively judged by others, guilt regarding DV, or the lack of personal resources to either leave the home or change the situation (9, 11, 12). This is not surprising given that Iran’s society is still dominated by traditional values including a patriarchal family structure and the social requirement that women should accept and tolerate DV in silence (10). However, incidence rates of DV in Iran are likely to be underreported due to expectations that women maintain secrecy about DV. Lack of knowledge about women’s legal rights and their shame in reporting the abuse to police are some of the most important hindrances (13).

At the individual level, these factors might include sociodemographic characteristics, such as women’s age, education, employment, health status, and experience of violence in the family of origin or present extended family household (11). A qualitative analysis of battered women found that women’s primary objective was to find ways of achieving non-violence rather than ending the relationship (12). Private strategies used by women to manage and reduce the violence include deciding to remain in the relationship while disengaging emotionally from the partner, remaining vigilant of the partner’s behavior and planning to do something only when the violence becomes more serious, resisting or fighting back, placating the partner, avoiding him, or actively silencing themselves (3, 12). However, researches show that DV is one of the main reasons given by couples seeking divorce (14, 15) and more than half of cases entering divorce mediation report having experienced DV (16-18). Also, research suggests that 38% to 43% of women with DV separate or divorce after a 2-year follow-up (19). A question that is posed here is: what personality traits that can influence the decision to remain in the relationship or to ask for a divorce? To answer this question, we studied the effect of two personality traits: self-concept and self-differentiation.

Self-concept defines how we value ourselves. In general, self-concept can be defined as an organized entity of characteristics, features, standpoints, emotions, images and abilities that the person ascribes to himself or herself. These psychological entities form the so-called referential frame with which an individual coordinates and orients his or her behavior (20). Thus, self-concept is a psychological construct through which we perceive ourselves, the way we see ourselves and the way others see us, and how we behave accordingly. Self-concept develops during our interactions with others. The concept also comprises the ability to think about what we are likely to do in a given situation (20, 21). Self-concept refers to an individual’s perception of self in relation to characteristics such as academics, gender roles, sexuality, communication with other people and racial identity (20, 22). Self-concept develops over a lifetime and varies among individuals. As defined by Bracken (1992), self-concept is a learned response pattern that reflects the individual’s evaluation of past behavior and experiences and predicts the individual’s future behavior (22). Also, Differentiation of self is an important familial factor that has been found to be related to stress and anxiety (23) .Differentiation of self is the internal, psychic condition of being able to distinguish feelings from thoughts. Differentiation is described on a continuum where at one end (high differentiation) an individual is able to maintain a strong sense of self in the midst of uncertain circumstances and intense emotional relationships. At the other end of the continuum (low differentiation), a person loses self in situations that produce anxiety, becoming emotionally dependent and enmeshed or fused psychologically with others (24). Bowen believed that the level of differentiation persons achieve in their family of origin has an important and lasting effect on their life (25). Also, studies have shown that differentiation of self has been positively correlated with psychological well-being, ethnic belongingness, self-control, and marital adjustment; and negatively correlated with chronic anxiety, psychiatric symptoms, fears of abandonment, and desire for merger (25). More differentiated persons are expected to establish greater autonomy in a relationship without experiencing debilitating fears of abandonment and to achieve emotional intimacy in that same relationship without fear of feeling smothered. It is also the case that those with higher levels of differentiation are expected to act from their values and sense of self rather than be influenced by the emotional reactivity of others. Thus, a more differentiated partner will be able to separate out the anxiety due to symptoms and will be able to act toward the other in ways that would maintain relationship satisfaction and be supportive of one another (26). Bowen says that low differentiation levels contribute to marital conflicts. Haber (27), for example, found that couples with higher levels of differentiation had lower levels of relationship conflicts. Another study of married couples also found a significant relationship between differentiation and marital satisfaction. In a similar vein, Skowron (28) found a positive correlation between differentiation and marital satisfaction, with husbands’ emotional cut-off scores particularly correlating with both husbands’ and wives’ marital satisfaction scores. The study showed that the couples who have low levels of differentiation and high levels of emotional reactivity, and cut off and fusion with others experience higher levels of stress and anxiety in their relationships. These couples are less satisfied with marital too (29). As mentioned above, previous researches have shown that DV has a deteriorating influence on society by affecting victims, their children, families and friends, as well as the social and financial relationships. In fact, several researches and studies have been performed on the factors affecting DV such as socio-demographic characteristics, psychological and cultural factors. In this research, we examined the personality characteristics that influence the decision to remain in this destructive relationship.

## 2. Objectives

According to above contents, the current study aims to determine and compare between aspects of self-differentiation and self-concept in divorced and non-divorced women.

## 3. Materials and Methods

### 3.1. Participants and Plan

This study was conducted in 2011 in Tehran; the present study is causal-comparative study. The sample consisted of two groups: 80 divorced women who experience DV and a control group: The sample of divorced women group was chosen with convenience sampling in “State Family Court 2”.

The control group consisted of 80 volunteers women who experienced DV matched group for age and level of education. The sample of this group was chosen by convenience sampling in psychology center. Age ranged from 15 to 40 years. The inclusion criteria were as follow: 1- Age ranged from 15 to 50 years. 2- High school as the minimum level of education. 3- Have experienced DV. 4-without any severe mental and physical illnesses. 5- Less than 12 months have passed since obtaining the divorce.

### 3.2. Measurements

A socio-demographic data sheet was used to record personal information of the participants including age, education, marriage duration and psychiatric diagnosis.

Differentiation of Self Inventory (DSI): This inventory is a 45-item self-report measure that focuses on adults, their significant relationships, and their current relations with family of origin. Participants respond to items on a six-point Likert-type scale, ranging from one (not at all true for me) to six (very true for me). The DSI contains four subscales: emotional reactivity (ER; 11 items), I position (IP; 11 items), emotional cutoff (EC; 12 items) and fusion with others (FO; 12 items). The DSI full-scale score was calculated by reversing raw scores on all items on the ER, EC, and FO sub-scales and one item (35) on the IP sub-scale and totaling them, so that higher scores reflected greater differentiation (less emotional reactivity, less difficulty in maintaining I-positions, less emotional cut off and less fusion). The original study reported internal consistency reliabilities (Cronbach’s alpha) as follows: DSI full-scale = 0.73, ER = 0.66, IP = 0.65, EC = 0.73 and FO = 0.60. Internal consistency reliabilities for the Persian version of the scale are reported as well: DSI full scale = 0.94, ER = 0.78, EC = 0.86, IP = 0.83 and FO = 0.88.

Carl Rodgers' self-concept test: The self-concept test includes two forms of A and B. The A form estimates the attitude of one about the real self and the B form estimates the attitude of one about the ideal self. In both forms, a collection of 25 pairs of opposite personal characteristics is presented. The test taker first identifies what he considers himself in relation to every characteristic in form A; however, in form B, he considers his situation in relation to every pair of opposite characteristics in the table based on his dreams and ideals between numbers 1 to 7. After estimating the individuals' self-concept score based on the test instruction, a score between 0 to 7 shows positive self-concept, a score between 7.01 to 10 shows a negative self-concept and a score above10.01 shows nor tic self-concept. The correlation coefficient between forms A and B is 82% suggesting the validity of the questionnaires and the reliability of the test was reported to be 57%.

### 3.3. Procedure

All participants completed 3 questionnaires, including Socio-demographic data sheet, Carl Rodgers' self-concept, Differentiation of Self Inventory (DSI) questionnaire. Then, the collected data was analyzed with SPSS-16 software. Data was analyzed between the two groups using independent t- test for two groups.

## 4. Results

In Table 1, the results of socio-demographic characteristics of all of the participants are indicated. As shown in Table 1, the higher category of age belonged to 21-30 and high level of education was for the baccalaureate degree. Also, 45 percent of participants were householder and 55 percent of them were employed and high duration of marriage belonged to 1-3 years. Regarding the results of Table 2, the t- test indicated that there was a significant difference between the items of Emotional reactivity, I position and Emotional cut off, Fusion with others, Differentiation of self-total, Self-concept (P < 0.01). The comparison of average of scores between two groups indicated that the divorced group had the highest score in all sub-scales. 

**Table 1.** Socio-Demographic Characteristic of all Participants in Percent

	n_i_^*^	Cf^*^
**Age group, year**		
15-20	22	13.8
21-30	76	61.2
31-40	42	78.5
41-50	20	100
**Duration of marriage**		
Less than 1	37	23.1
1-3	42	49.3
4-5	27	66.2
6-10	26	82.5
Higher than 10	28	100
**Education**		
High School	10	6.2
Diploma	44	33.8
Baccalaureate Degree	92	91.2
Master’s	14	100
**Job status**		
Householder	72	45
Employed	88	100

^*^Abbreviations: n_i_, absolute frequency; Cf, cumulative frequency

**Table 2.** Mean, SD and P Value of Total and Subscales of Self- Differentiation and Self-Concept for Divorced (Group 1, N=80) and Non-Divorced (Group 2, N=80) Groups

	Mean ± SD^*^	df^*^	P Value	t^*^
**Emotional reactivity**		140.22	0.001	13.67
Group 1	40.66 ± 3.47			
Group 2	31.3 ± 5.04			
**I position**		132.09	0.001	10.62
Group 1	40.86 ± 3.24			
Group 2	33.56 ± 5.22			
**Emotional cut off**		140.27	0.001	19.74
Group 1	48.4 ±3.88			
Group 2	33.27 ± 5.64			
**Fusion with others**		158	0.001	15.64
Group 1	43.91 ± 6.4			
Group 2	29.41 ± 5.27			
**Differentiation of self total**		132.26	0.002	22.64
Group 1	173.84 ± 9.67			
Group 2	127.55 ± 15.52			
**Self-concept**		158	0.000	-5.55
Group 1	11.38 ± 2.94			
Group 2	13.57 ± 1.16			

^*^Abbreviations: SD, standard deviation; df, degree of freedom; t, Student's t–test

## 5. Discussion

This study was designed to compare self-differentiation and self-concept in divorced and non-divorced women who experience DV. As shown in Table 2, the average of self-differentiation of divorced group (173/84) is more than non-divorced group (127/55). So, the findings of previous study indicated that higher levels of differentiation were associated with lower levels of psychological symptoms and perceived stress. Highly differentiated individuals exhibited reflective coping (e.g. identifying the cause of emotions to solve problems) while poorly differentiated individuals exhibited suppressive (e.g. avoidance) and reactive (e.g. impulsive decisions) coping. (20, 30) Highly differentiated individuals were able to quickly achieve emotional balance after eliminating the stress (31). Also, self-differentiation affects the marital adjustment and previous researchers have found a negative correlation between differentiation of self and depression (26, 32) Skowren examined the utility of Bowen family systems theory for predicting quality of marital life. In particular, the results showed that emotional cutoff and emotional reactivity predicted marital discord (28). Research has established the relationship between the quality of marital relationship, indicated by intimacy and love, and psychological well-being of the individual (33). In marital relationship, whenever the differentiation level falls low, fusion takes place between couples, leading to low marital quality and compatibility (39). On the other hand, Self-differentiation factor affects the incidence of divorce. As in the study, there are significant differences between self-differentiation of divorced applicants and those who were willing to continue the marriage. The research examined the relationship between self-differentiation and emotional intelligence in divorce applicants. Between the two groups of non-clinical and divorced applicants, there is a significant difference in Emotional reactivity, I position, emotionally cut off, self-differentiation and emotional intelligence (34). Poorly differentiated individuals would display a lack of stability, low tolerance for individuality, poor intimacy among couple, anxiety, and rigid behaviors. According to Papero (2000), well-differentiated individuals make their decisions and actions based on well-developed sets of internal beliefs and principles that they have thought about carefully and tested in real situations in their lives. Therefore, their behaviorstolerate differences in others without intense reactive pressure, and thus, they can continue to make their decisions and accept responsibility for the outcomes without blaming others, seeing themselves as victims, or being controlled by others (35). Hence, highly differentiated women, with relying on the distinction between intellect and emotion and their distinction from cultural context, have great ability to make the decision to remain or divorce. However, the poorly differentiated women, passively continue to live with violence due to fear for themselves, fear of losing their children or fear of being negatively judged by others (12). 

According to Table 2, the divorced group has more positive self-esteem than the non-divorced group. Some studies have considered the variables of self-concept as factors that may aid in selecting coping strategies. One of these variables is self-concept clarity which was related to behavior in stressful situations. It is contributed positively to active coping styles such as planning and taking action and contributed negatively to passive coping styles such as denial (12). Köhlmeier and Amann (2006) have shown the influence of sense of coherence and self-concept on coping with DV (36). Also, Kyung Mi Sung (2011) reported that in the early adolescents of the Korean female, positive self-concept was highly correlated with some approach coping strategies such as positive reappraisal and problem solving, and negative self-concept was correlated with some avoidance coping strategies such as acceptance/resignation and emotional discharge (12). Several studies have found that women with low self-esteem appear to be more willing to forgive their offenders than are women with high self-esteem (37, 38). Results of current study is in line with previous study show that women with low self-esteem may define themselves more in terms of their relations with others and are therefore less willing to relinquish those relationships, even in the case of offenses that include interpersonal violence. Conversely, women who have high self-esteem are not only less inclined to define their self-worth in terms of their relations with others, but they may also be more likely to interpret any offense as inconsistent with their perceived self-worth and therefore as a more serious affront. Women with high self-esteem may hold strong feelings about respecting and valuing themselves. Previous researches indicate that anger, resentment, and hatred are legitimate emotions that one will likely experience after being injured by another. To assume that these emotions are always detrimental to the injured person is a mistake because these emotions may help to protect important values (e.g. self-respect, self-defense, moral order) that are important to an individual (38). It would appear that women with DV and high self-concept disappoint to change the behavior of their partner after frequent efforts for changing the status quo. So, they decide to leave their abuse relationship due to the effects of divorce on their children and themselves. One of the limitations of this study is the lack of sufficient cooperation among the relevant institutions such as the courts and counseling centers. According to Lotfabadi, about one third of the investigated Iranian mothers reported some type of DV (10). Hence, it is suggested that according to the results of the first hypothesis, it should be restudied and compared to those without a domestic violence. Since DV is resulting from the dysfunctional interaction between family member, hence positive self-concept and highly self-differentiation of one of the partner is not sufficient to maintain their family intact. So that it suggests premarital education includes interpersonal skills such as self-differentiation and self-concept. 
